# Complete genome sequence of *Mycobacterium tuberculosis* K from a Korean high school outbreak, belonging to the Beijing family

**DOI:** 10.1186/s40793-015-0071-4

**Published:** 2015-10-14

**Authors:** Seung Jung Han, Taeksun Song, Yong-Joon Cho, Jong-Seok Kim, Soo Young Choi, Hye-Eun Bang, Jongsik Chun, Gill-Han Bai, Sang-Nae Cho, Sung Jae Shin

**Affiliations:** Department of Microbiology, Brain Korea 21 PLUS Project for Medical Science, Yonsei University College of Medicine, Seoul, South Korea; Institute for Immunology and Immunological Diseases, Yonsei University College of Medicine, Seoul, South Korea; ChunLab Inc., Seoul National University, Seoul, South Korea; Korean Institute of Tuberculosis, Korean National Tuberculosis Association, Osong, South Korea

**Keywords:** *Mycobacterium tuberculosis*, Korean Beijing strain, Outbreak, *M. tuberculosis* K complete genome, TB clinical strain, TB Beijing family

## Abstract

*Mycobacterium tuberculosis* K, a member of the Beijing family, was first identified in 1999 as the most prevalent genotype in South Korea among clinical isolates of *M. tuberculosis* from high school outbreaks. *M. tuberculosis* K is an aerobic, non-motile, Gram-positive, and non-spore-forming rod-shaped bacillus. A transmission electron microscopy analysis displayed an abundance of lipid bodies in the cytosol. The genome of the *M. tuberculosis* K strain was sequenced using two independent sequencing methods (Sanger and Illumina). Here, we present the genomic features of the 4,385,518-bp-long complete genome sequence of *M. tuberculosis* K (one chromosome, no plasmid, and 65.59 % G + C content) and its annotation, which consists of 4194 genes (3447 genes with predicted functions), 48 RNA genes (3 rRNA and 45 tRNA) and 261 genes with peptide signals.

**Electronic supplementary material:**

The online version of this article (doi:10.1186/s40793-015-0071-4) contains supplementary material, which is available to authorized users.

## Introduction

*Mycobacterium tuberculosis*, the bacterium responsible for causing tuberculosis, carries the world record for the highest mortality as a single infectious agent. According to a 2014 World Health Organization report, 8.6 million people were estimated to be new TB cases, and approximately 1.3 million people died from TB worldwide [1]. Strains of *M. tuberculosis* in different geographical locations or populations may have different levels of virulence due to co-evolutionary processes, which consequently leads to varying epidemiological dominance [2, 3].

Among the various *M. tuberculosis* strains, *M. tuberculosis* strains belonging to the Beijing genotypes are more prone to induce disease progression and relapse from the latent state [4, 5]. For example, HN878, the causative agent of major TB outbreaks in Texas prisons between 1995 and 1998 [6], belongs to the W-Beijing family, which expresses a highly biologically active lipid species (phenolic glycolipid). HN878 causes rapid progression to death in mice compared to other clinical isolates (CDC1551) or standard laboratory-adapted virulent strains (H37Rv^T^) [7]. In addition, the Beijing genotypes are associated with greater drug resistance than the other *M. tuberculosis* genotypes [8]. The frequency of Beijing *M. tuberculosis* is estimated to be 85 to 95 % in South Korea [9]. Recently, the Beijing strains have spread all over the world, including the US, Europe, and Africa, and account for over 13 % of all of the *M. tuberculosis* strains worldwide [10–12].

Unusually high rates of pulmonary TB occurred in senior high schools in Kyunggi Province in South Korea in 1998 [13]. During the national survey for genotyping analysis of clinical *M. tuberculosis* isolates in 1999, a single strain with a unique restriction fragment length polymorphism (RFLP) profile was the most frequently identified strain [13]. This particular *M. tuberculosis* K strain phylogenetically belongs to the Beijing genotype and is the most dominant *M. tuberculosis* strain in South Korea.

*M. tuberculosis* K replicates rapidly during the early stages of infection in a murine model of TB, causing a more severe pathology and a high level of reactivation from latent infection [14]. In addition, the Bacillus-Calmette-Guérin vaccination is less effective against an *M. tuberculosis* K infection than H37Rv^T^ (unpublished data). These remarkable features of *M. tuberculosis* K are associated with its high transmissibility and dominance in South Korea. However, the molecular mechanisms of virulence and the pathogenicity-related genetic features of this strain remain unclear. To understand the genomic features of the strain in detail, we sequenced and annotated the complete genome of *M. tuberculosis* K.

## Organism information

### Classification and features

**Fig. 1:**
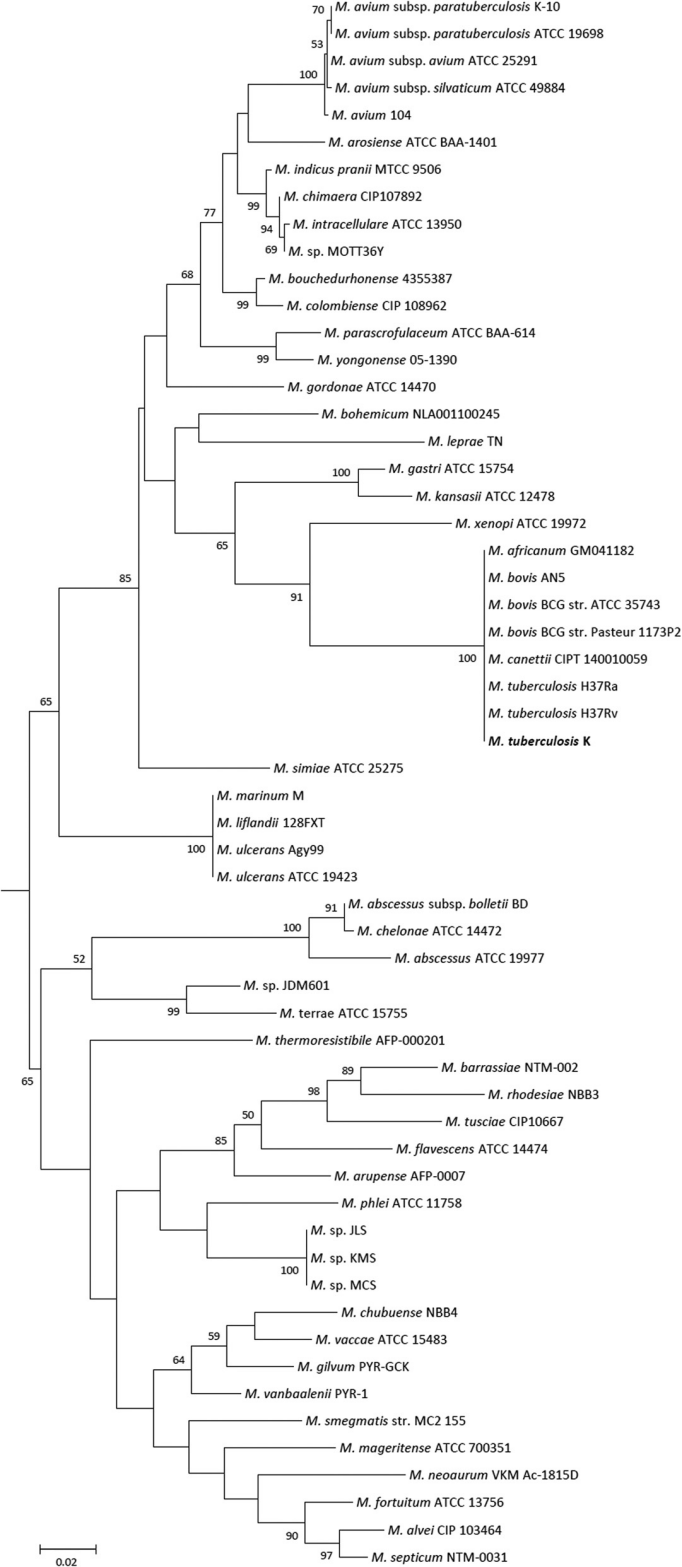
Phylogenetic tree showing the relationships of *M. tuberculosis* K with other *Mycobacterium* species based on aligned sequences of the *rpoB* gene. 711 bp internal region was used for phylogenetic analysis. All sites were informative and there were no gap-containing sites. Phylogenetic tree was built using the Maximum-Likelihood method based on Tamura-Nei model by MEGA. Bootstrap analysis [37] was performed with 500 replicates to assess the support of the clusters. Bootstrap values over 50 are shown at each node. The bar represents 0.02 substitutions per site

A representative genomic *rpoB* gene of *M. tuberculosis* K was compared with those obtained using BLASTN [15] with the default settings (only highly similar sequences). The sequence of the single *rpoB* gene copy was found in the genome. The *rpoB* gene, which was derived from the *M. tuberculosis* K genome sequence, showed 99.97 % sequence similarity to the *M. tuberculosis* H37Rv^T^ that was deposited in GenBank (GenBank accession: CP007803.1). We identified only one single-nucleotide polymorphism within the entire rpoB gene (3519 bp) in *M. tuberculosis* K compared to *M. tuberculosis* H37Rv^T^ (C3225T). *M. tuberculosis* K shares a high nucleotide sequence similarity with *M. tuberculosis* H37Rv^T^ and other mycobacteria (Table 1, Fig. 1 and Additional file 1: Table S1). Figure 1 shows the phylogenetic position of *M. tuberculosis* K in the partial *rpoB*-based tree. For a more detailed analysis, the whole-genome sequences were used for an average nucleotide identity analysis (Additional file 2: Figure S1). The ANI results showed that *M. tuberculosis* K belongs to the *M. tuberculosis* group but is separated from the other *M. tuberculosis* strains. The 16S rRNA gene sequence of *M. tuberculosis* K showed 100 % similarity with *M. tuberculosis* H37Rv^T^.

**Table 1:** Classification and general features of *M. tuberculosis* K according to the MIGS recommendation [16]

MIGS ID	Property	Term	Evidence code^a^
	Classification	Domain *Bacteria*	TAS [26]
		Phylum *Actinobacteria*	TAS [27]
		Class *Actinobacteria*	TAS [28]
		Order *Actinomycetales*	TAS [28–31]
		Family *Mycobacteriaceae*	TAS [28–30, 32]
		Genus *Mycobacterium*	TAS [30, 33, 34]
		Species *Mycobacterium tuberculosis*	TAS [35]
		Strain K (CP007803.1)	
	Gram stain	Weakly positive	TAS [35]
	Cell shape	Irregular rods	TAS [35]
	Motility	Non motile	TAS [35]
	Sporulation	Nonsporulating	NAS
	Temperature range	Mesophile	TAS [35]
	Optimum temperature	37 °C	TAS [35]
	pH range; Optimum	5.5–8; 7	IDA
	Carbon source	Asparagine, Oleic acid, Potato starch	TAS [14, 35]
MIGS-6	Habitat	Human-associated: Human lung	TAS [35]
MIGS-6.3	Salinity	Normal	TAS [35]
MIGS-22	Oxygen	Aerobic	TAS [35]
MIGS-15	Biotic relationship	Free-living	NAS
MIGS-14	Pathogenicity	Hypervirulent	TAS [14, 35]
	Biosafety level	3	NAS
	Isolation	Sputum of TB patient	TAS [35]
MIGS-4	Geographic location	High schools in Kyunggi Province, Republic of Korea.	TAS [35]
MIGS-5	Sample collection time	1999	TAS [35]
MIGS-4.1	Latitude Longitude	37.274377	NAS
MIGS-4.2	Longitude	127.009442	NAS
MIGS-4.4	Altitude	Not reported	NAS

Evidence codes - IDA: Inferred from Direct Assay; TAS: Traceable Author Statement (i.e., a direct report exists in the literature); NAS: Non-traceable Author Statement (i.e., not directly observed for the living, isolated sample, but based on a generally accepted property for the species, or anecdotal evidence). These evidence codes are from the Gene Ontology project [36]

**Fig. 2:**
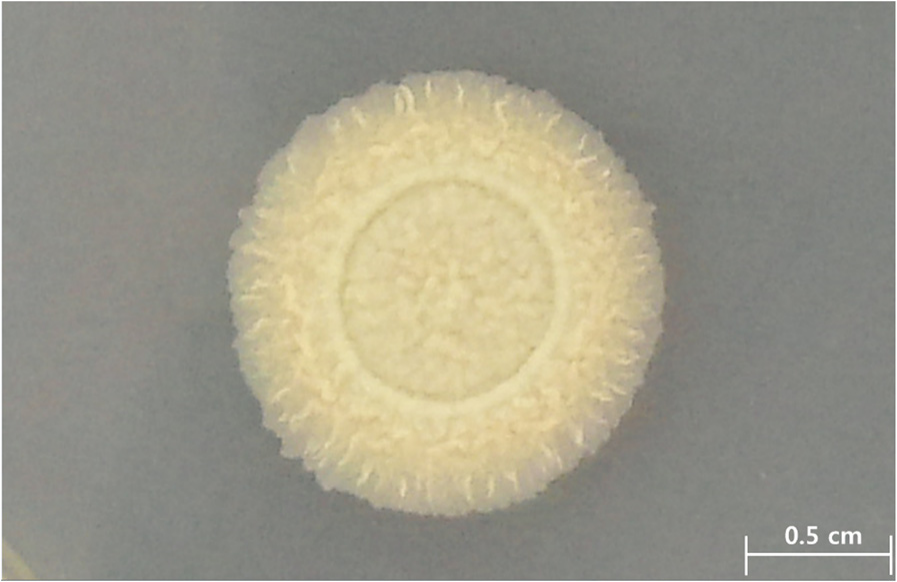
Image of *Mycobacterium tuberculosis* K using the appearance of colony morphology on 7H10-OADC solid medium

**Fig. 3:**
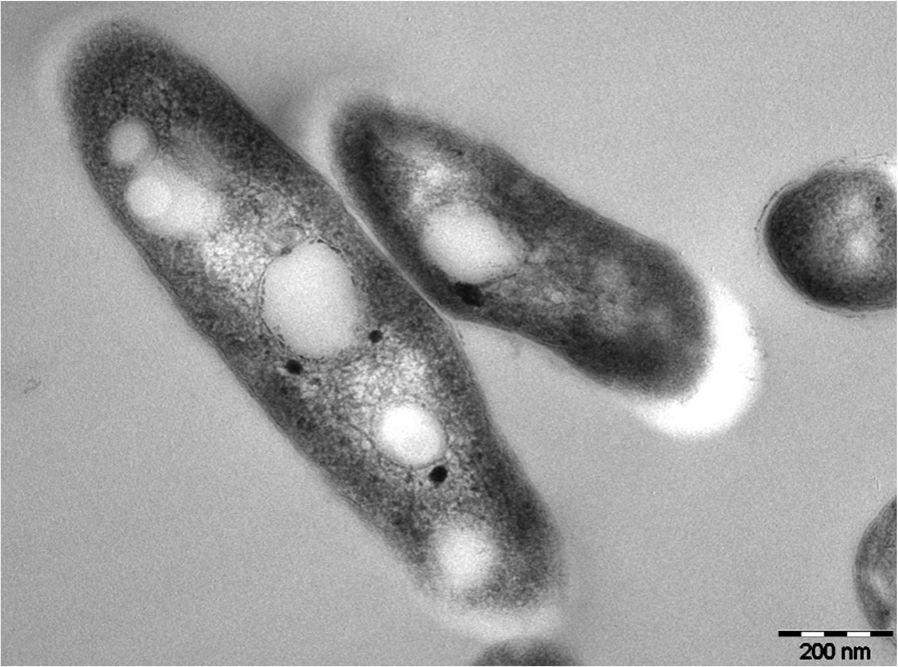
Transmission electron microscopy of *Mycobacterium tuberculosis* K

*M. tuberculosis* K is an aerobic, non-motile rod with a cell size of approximately 0.2–0.5 × 1.0–1.5 μm. It stains weakly positive under Gram staining and contains lipid bodies (Fig. 2). The colonies are slightly yellowish and appear rough and wrinkled on a 7H10-OADC plate (Fig. 3). The viable temperature range for growth is 4–37 °C, with optimum growth at 30–37 °C. The viable pH range is 5.5–8.0, with optimal growth at pH 7.0–7.5.

*M. tuberculosis* K is resistant to ampicillin, penicillin, chloramphenicol, erythromycin, azithromycin, clarithromycin and tetracycline, but it is susceptible to rifampicin, isoniazid, pyrazinamide, ethambutol, cycloserine, protionamide, amikacin, capreomycin, kanamycin, streptomycin, moxifloxacin, levofloxacin and ofloxacin. To investigate the phenotype of *M. tuberculosis* K, we observed 10^6^*M. tuberculosis* K bacilli under transmission electron microscopy. Briefly, the immobilized bacteria were rinsed with phosphate-buffered saline and fixed in 2.0 % paraformaldehyde and 2.0 % glutaraldehyde in 1x PBS with 3 mM MgCl_2_ (pH 7.2) for at least 1 h at room temperature. The bacterial cells were transferred to propylene oxide and were gradually infiltrated with Spurr’s low-viscosity resin (Polysciences, Warrington, USA): propylene oxide. After three changes in the 100 % Spurr’s resin, the pellets were cured at 60 °C for two days. The sections were cut on an ultramicrotome using a Diatome Diamond knife (Electron Microscopy Sciences, Hatfield, USA). Eighty-nanometer sections were picked up on formvar-coated 1 × 2-mm copper slot grids and stained with tannic acid and uranyl acetate followed by lead citrate. The grids were examined and photographed using TEM (JEM-1011, JEOL, Japan).

## Genome sequencing information

### Genome project history

*Mycobacterium tuberculosis* K and the other K-related strains comprise the most dominant genotype of *M. tuberculosis* in South Korea, but the genomic characteristics and genetic information regarding this strain are still poorly understood. This organism was selected to gain understanding of the molecular pathogenesis of the highly pathogenic and prevalent strain of *M. tuberculosis* in South Korea.

As the reference strain for studying tuberculosis in Korea, in this study, *M. tuberculosis* K was selected and sequenced. We used two different next-generation sequencing methods: Sanger and Illumina. The Sanger sequencing was performed at the Korea Research Institute of Bioscience and Biotechnology, Daejeon, South Korea. The NGS sequencing, finishing and genome annotation was performed by ChunLab Inc., Seoul, Korea, and the finished genome sequence and the related data were deposited in GenBank under the accession number CP007803.1. Table 2 presents the project information and its association with MIGS version 2.0 compliance [16].

**Table 2:** Project information

MIGS ID	Property	Term
MIGS-31	Finishing quality	Finished
MIGS-28	Libraries used	Three genomic libraries: two Sanger libraries; 2 kb shotgun library, fosmid library, respectively and one Illumina library
MIGS-29	Sequencing platforms	Sanger, Illumina MiSeq 250 bp paired-end
MIGS-31.2	Fold coverage	8.3x (Sanger), 551.66x (Illumina)
MIGS-30	Assemblers	Phred/Phrap/Consed, CLC genomics workbench v6.5, CodonCode Aligner v3.7
MIGS-32	Gene calling method	Glimmer v 3.02
	Locus Tag	MTBK
	Genbank ID	CP007803.1
	Genbank Date of Release	June 05, 2014
	GOLD ID	Gp0032286
	BIOPROJECT	PRJNA178919
MIGS-13	Source Material Identifier	The Korean Institution of Tuberculosis
	Project relevance	Human-associated pathogen

### Growth conditions and genomic DNA preparation

*M. tuberculosis* K was kindly provided by the Korean Institute of Tuberculosis, Seoul, Korea. *M. tuberculosis* H37Rv^T^, which is stored at the International Tuberculosis Research Centre (ITRC, Masan, South Korea), was also used in this study. *M. tuberculosis* was cultured aerobically at 37 °C in Middlebrook 7H10 media containing 0.02 % glycerol and 10 % OADC for 4 weeks.

From the *M. tuberculosis* cultures grown in the 7H10 media for a month, the bacterial DNA was isolated as previously described [17]. In short, the bacilli in suspension were killed by heating at 80 °C for 30 min, and after centrifugation, the cell pellets were resuspended in 500 μl of TE buffer (0.01 M Tris–HCl, 0.001 M EDTA [pH 8.0]). The cells were treated with lysozyme (1 mg/ml) for 1 h at 37 °C, then with 10 % sodium dodecyl sulfate (SDS) and proteinase K (10 mg/ml) for 10 min at 65 °C prior to the DNA isolation. A total of 80 μl of N-acetyl-N,N,N,-trimethyl ammonium bromide was then added to approximately 500 μl of the lysed cell suspension, and the suspension was vortexed briefly and incubated for 10 min at 65 °C. An equal volume of chloroform-isoamyl alcohol (24:1, vol/vol) was added, and the mixture was vortexed for 10 s. The solution was then centrifuged for 5 min, and 0.6 volumes of isopropanol were added to the supernatant to precipitate the DNA. After cooling for 30 min at 20 °C, the DNA solution was centrifuged for 15 min, and the pellet was washed once with 70 % ethanol. Finally, the air-dried pellet was redissolved in 50 μl of 0.1x TE buffer and stored at −20 °C until use.

### Genome sequencing and assembly

The *M. tuberculosis* K genome was sequenced at KRIBB (Daejeon, South Korea) and ChunLab Inc. (Seoul, South Korea) using two Sanger libraries (2 kb random shotgun library and fosmid library) and one Illumina library. The random shotgun and fosmid libraries were prepared using the pTZ19U vector and the CopyControl Fosmid Library Production Kit (Epicentre, Madison, USA), respectively. For the Illumina sequencing, the genomic DNA was fragmented using dsDNA fragmentase (NEB, Hitchin, UK) to make it to the proper size for the library construction. The resulting DNA fragments were processed using the TruSeq DNA Sample Preparation Kit v2 (Illumina, Inc., San Diego, USA) following the manufacturer’s instructions. The final library was quantified using a Bioanalyzer 2100 (Agilent, Santa Clara, USA), and the average library size was 300 bp.

The genomic libraries were sequenced via Sanger sequencing on an ABI3730 and an Illumina MiSeq (Illumina, Inc., San Diego, USA). The generated Sanger sequencing reads (70,889 reads, total read length: 36,413,063 bp) and the Illumina paired-end sequencing reads (10,493,598 reads, total read length: 2,419,306,885 bp) were assembled using the Phred/Phrap/Consed package and CLC Genomics Workbench v6.5 (CLC bio, Aarhus, Denmark). The resulting contigs from the Sanger sequencing of the 2 kb random shotgun library were scaffolded by sequencing the reads from the fosmid clones, and the gaps in the scaffolds were closed using PCR and Sanger sequencing. The contigs and the Sanger sequence reads for the gap closure were combined via manual curation using Phred/Phrap/Consed and CodonCode Aligner 3.7.1 (CodonCode Corp., Centerville, USA). The final genome sequence was reviewed by remapping with the Illumina raw reads and correcting the dubious regions and errors.

### Genome annotation

The coding sequences were predicted by Glimmer 3.02 [18]. The tRNAs were identified using tRNAScan-SE [19], and the rRNAs were searched using HMMER with the EzTaxon-e rRNA profiles [20, 21]. The predicted CDSs were compared to catalytic families and NCBI Clusters of Orthologous Groups using rpsBLAST and the NCBI reference sequences SEED, TIGRFam, Pfam, Kyoto Encyclopedia of Genes and Genomes, COG and InterPro databases, using BLASTP and HMMER for the functional annotation [22–25]. Additional analyses and functional annotations for the genome statistics were performed using the Integrated Microbial Genomes platform.

## Genome properties

**Fig. 4:**
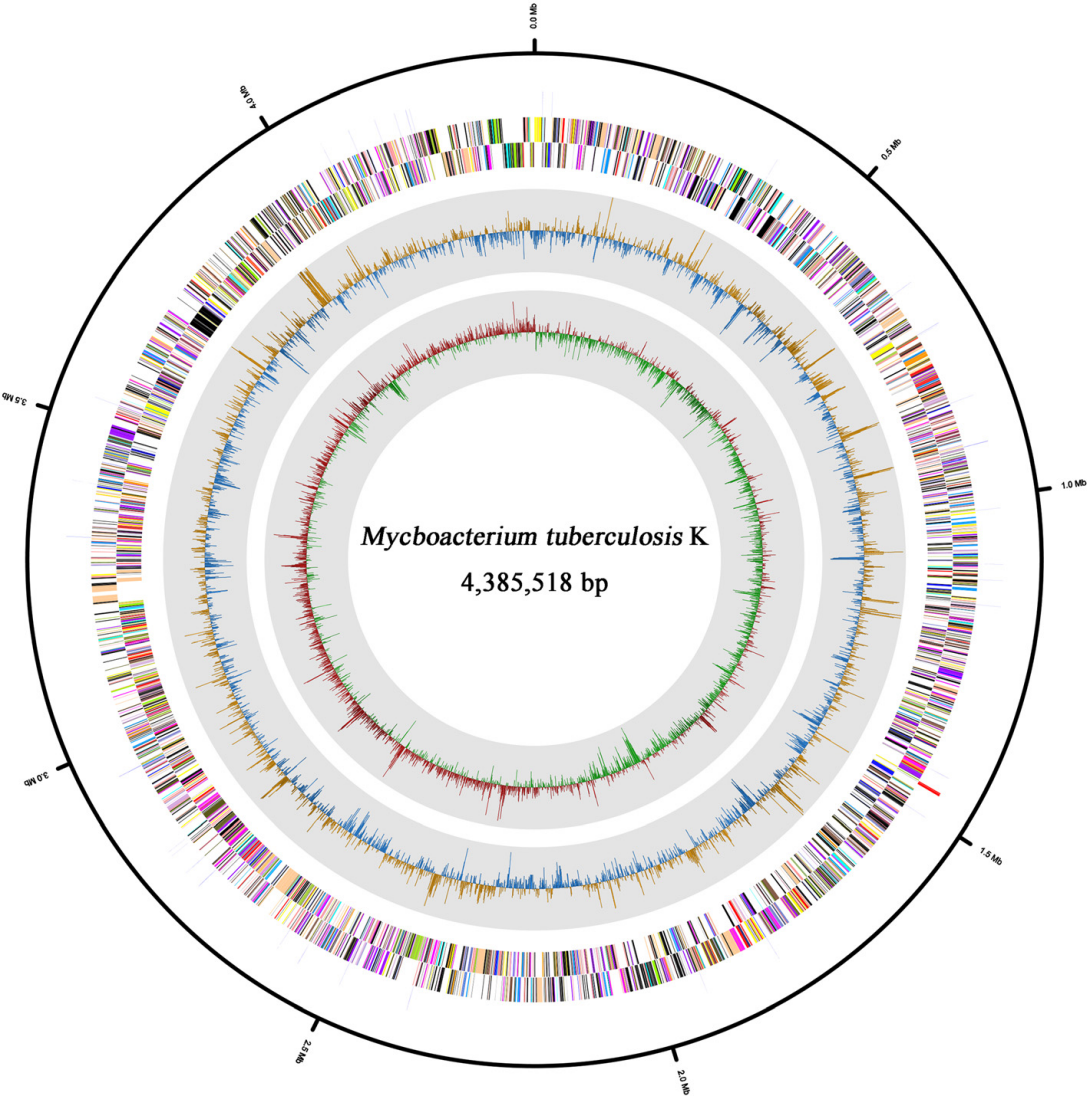
Graphical circular map of *M. tuberculosis* K strain genome. From the outside to the center: RNA features (ribosomal RNAs are colored as *blue*, and transfer RNAs are colored as *red*), genes on the forward strand and the reverse strand (colored according to the COG categories). The inner *two circles* show the GC ratio and GC skew. The GC ratio and GC skew are shown in *orange* and *red* indicates positive, and in *blue* and *green* indicates negative, respectively

The total length of the complete genome sequence was 4,385,518 bp, and no plasmid was found. The G + C content was determined to be 65.59 %, which is similar to other *M. tuberculosis* strains (65–66 %) (Fig. 4 and Table 4). Based on the gene prediction results, 4194 CDSs were identified, and 45 tRNAs and 1 rRNA operon were annotated. The total length of the genes was 3,953,484 bp, which makes up 90.15 % of the entire genome. The majority of the genes (82.19 %) were assigned putative functions, while the remaining genes (17.81 %) were annotated as hypothetical. A total of 2610 CDSs were assigned to functional COG groups, 3349 genes were assigned to Pfam domains and 261 genes had signal peptides. The genome properties and statistics are summarized in Table 3. The distributions of the genes among the COG functional categories are shown in Tables 4 and 5.

**Table 3:** Summary of genome: one chromosome and two plasmids

Label	Size (Mb)	Topology	INSDC identifier	RefSeq ID (Optional)
Chromosome	4,385,518	Circular	GenBank	CP007803.1

**Table 4:** Nucleotide content and gene count levels of the genome

Attribute	Value	% of total
Genome size (bp)	4,385,518	100.00
DNA coding (bp)	3,954,282	90.17
DNA G + C (bp)	2,876,511	65.59
DNA scaffolds	1	100.00
Total genes	4,194	100.00
Protein coding genes	4,146	98.86
Pseudo genes	2	0.05
RNA genes	48	1.14
Genes in internal clusters	NA	NA
Genes with function prediction	2,885	68.79
Genes assigned to COGs	2,892	69.74
Genes with Pfam domains	3,347	79.80
Genes with signal peptides	233	5.56
Genes coding transmembrane helices	810	19.31
CRISPR repeats	4	0.10

The total is based on either the size of the genome in base pairs or the total number of protein coding genes in the annotated genome

Also includes 1 pseudogene

**Table 5:** Number of genes associated with the 25 general COG functional categories

Code	Value	% age	COG category
J	193	4.66	Translation, ribosomal structure and biogenesis
A	10	0.24	RNA processing and modification
K	195	4.70	Transcription
L	106	2.56	Replication, recombination and repair
B	–	–	Chromatin structure and dynamics
D	37	0.89	Cell cycle control, cell division, chromosome partitioning
Y	–	–	Nuclear structure
V	84	2.03	Defense mechanisms
T	112	2.70	Signal transduction mechanisms
M	157	3.79	Cell wall/membrane/envelope biogenesis
N	8	0.19	Cell motility
Z	–	–	Cytoskeleton
W	1	0.02	Extracellular structures
U	23	0.55	Intracellular trafficking, secretion, and vesicular transport
O	117	2.82	Posttranslational modification, protein turnover, chaperones
C	195	4.70	Energy production and conversion
G	139	3.35	Carbohydrate transport and metabolism
E	186	4.49	Amino acid transport and metabolism
F	83	2.00	Nucleotide transport and metabolism
H	225	5.43	Coenzyme transport and metabolism
I	275	6.63	Lipid transport and metabolism
P	127	3.06	Inorganic ion transport and metabolism
Q	107	2.58	Secondary metabolite biosynthesis, transport and catabolism
R	246	5.93	General function prediction only
S	266	6.42	Function unknown
–	1254	30.26	Not in COGS

The total is based on the total number of protein coding genes in the annotated genome

## Conclusion

*M. tuberculosis* strains in different populations or geographical locations can exhibit different levels of virulence during the human-adaptation process with consequent varying epidemiological dominance. Importantly, clinical and epidemiological studies have demonstrated that the emergence of the Beijing strains may be associated with multi-drug resistance and a high level of virulence, resulting in increased transmissibility and rapid progression from infection to active disease. The *M. tuberculosis* K strain, which was isolated from an outbreak of pulmonary TB in senior high schools in South Korea, phylogenetically belongs to the Beijing genotype. Here, we present a summary classification and a set of genomic features of *M. tuberculosis* K together with the description of the complete genome sequence and annotation. The genome of the *M. tuberculosis* K strain is 4.4 Mbp with a GC content of 65.59 %. *M. tuberculosis* K genome contains several key virulence factors that are absent in the *M. tuberculosis* H37Rv^T^ genome, such as PE/PPE/PE-PGRS family proteins considered to be involved in granuloma formation and antigenic variations. Further functional analyses of the *M. tuberculosis* K-specific virulence factors involved in pathogenesis are currently under investigation. These studies may help us to understand the geographical evolution and molecular pathogenesis of this unique genotypic *M. tuberculosis*.

## Additional files

Additional file 1: Table S1. Associated MIGS record. (DOCX 36 kb) Additional file 2: Figure S1. The genome tree showing the relationships of *M. tuberculosis* K with other *Mycobacterium* species based on ANI values. Description of data: To convert the ANI into a distance, its complement to 1 was taken. From this pairwise distance matrix, an ANI tree was constructed using the UPGMA clustering method. (PDF 846 kb)

## Abbreviations

TB: Tuberculosis
TEM: Transmission electron microscopy
ANI: Average nucleotide identity
RFLP: Restriction fragments length polymorphism
