# Supplementary material for: Complete genome sequence of Mycobacterium tuberculosis K from a Korean high school outbreak, belonging to the Beijing family
Source: Stand Genomic Sci. 2015 Oct 14;10:78. doi: 10.1186/s40793-015-0071-4 (PMC4606834; doi:10.1186/s40793-015-0071-4)
Supplement: Additional file 1: Table S1. — Associated MIGS record. (DOCX 36 kb) [file 40793_2015_71_MOESM1_ESM.docx]

**Table S1.** Associated MIGS record

| **MIGS-ID** | Field name | Description |
| --- | --- | --- |
| **MIGS-1** | Submit to INSDC/Trace archives | CP007803.1 (chromosome) |
| **1.1** | PID | Not reported |
| **1.2** | Trace Archive | Not reported |
| **MIGS-2** | MIGS CHECK LIST TYPE | BA |
| **MIGS-3** | Project Name | Complete genome sequence of *Mycobacterium tuberculosis* K |
| **MIGS-4** | Geographic Location | High schools in Kyunggi Province South Korea |
| **4.1** | Latitude | 37.274377 |
| **4.2** | Longitude | 127.009442 |
| **4.3** | Depth | Not reported |
| **4.4** | Altitude | Not reported |
| **MIGS-5** | Time of Sample collection | 1999 |
| **MIGS-6** | Habitat (EnvO) | Human-associated: human-lung |
| **6.1** | temperature | 37°C |
| **6.2** | pH | 7.0-7.5 |
| **6.3** | salinity | Not reported |
| **6.4** | chlorophyll | Not reported |
| **6.5** | conductivity | Not reported |
|  |  |  |
| **6.6** | light intensity | Not reported |
| **6.7** | dissolved organic carbon (DOC) | Not reported |
| **6.8** | current | Not reported |
| **6.9** | atmospheric data | Not reported |
| **6.10** | density | Not reported |
| **6.11** | alkalinity | Not reported |
| **6.12** | dissolved oxygen | Not reported |
| **6.13** | particulate organic carbon (POC) | Not reported |
| **6.14** | phosphate | Not reported |
| **6.15** | nitrate | Not reported |
| **6.16** | sulfates | Not reported |
| **6.17** | sulfides | Not reported |
| **6.18** | primary production | Not reported |
| **MIGS-7** | Subspecific genetic lineage | *Mycobacterium tuberculosis* K |
| **MIGS-9** | Number of replicons | 1 |
| **MIGS-10** | Extrachromosomal elements | 0 |
| **MIGS-11** | Estimated Size | 4.39 Mb |
| **MIGS-12** | Reference for biomaterial or Genome report | NCBI BioProject – PRJNA178919 |
| **MIGS-13** | Source material identifiers | Middlebrook 7H10 |
| **MIGS-14** | Known Pathogenicity | Tuberculosis |
|  |  |  |
| **MIGS-15** | Biotic Relationship | Facultative intracellular pathogen |
| **MIGS-16** | Specific Host | Human |
| **MIGS-17** | Host specificity or range (taxid) | 9606 |
| **MIGS-18** | Health status of Host | Pulmonary tuberculosis in lungs |
| **MIGS-19** | Trophic Level | Not reported |
| **MIGS-22** | Relationship to Oxygen | Obligately aerobic |
| **MIGS-23** | Isolation and Growth conditions | Human sputum & 7H9+OADC |
| **MIGS-27** | Nucleic acid preparation | Laboratory protocol |
| **MIGS-28** | Library construction | Random shotgun library, fosmid library, Illumina MiSeq shotgun library |
| **28.1** | Library size | 2 kb pTZ19U, 40 kb pcc1Fos, 300 bp (Illumina) |
| **28.2** | Number of reads | 70,889 (3 kb Sanger), 4,034 (40 kb Sanger),10,493,598 (Illumina) |
| **28.3** | vector | pTZ19U |
| **MIGS-29** | Sequencing method | Sanger, Illumina MiSeq 250 bp paired-end |
| **MIGS-30** | Assembly | *de novo* assembly |
| **30.1** | Assembly method | Phred/Phrap/Consed, CLC genomics workbench v 6.5, CodonCode Aligner v 3.7 |
| **30.2** | estimated error rate | Not reported |
| **30.3** | method of calculation | Not reported |
| **MIGS-31** | Finishing strategy | The contigs were scaffolded by sequence reads from fosmid clones, and gaps were closed and confirmed with PCR and manual curation with CodonCode Aligner v 3.7 |
| **31.1** | Status | Finished |
| **31.2** | coverage | 551.66x |
| **31.3** | contigs | 1 |
| **MIGS-32** | Relevant SOPs | doi:10.4056/sigs.632 |
| **MIGS-33** | Relevant e-resources | http://www.ncbi.nlm.nih.gov/bioproject/PRJNA178919 |
