# Supplementary figures and images for: Complete genome sequence of Mycobacterium tuberculosis K from a Korean high school outbreak, belonging to the Beijing family
Source: Stand Genomic Sci. 2015 Oct 14;10:78. doi: 10.1186/s40793-015-0071-4 (PMC4606834; doi:10.1186/s40793-015-0071-4)

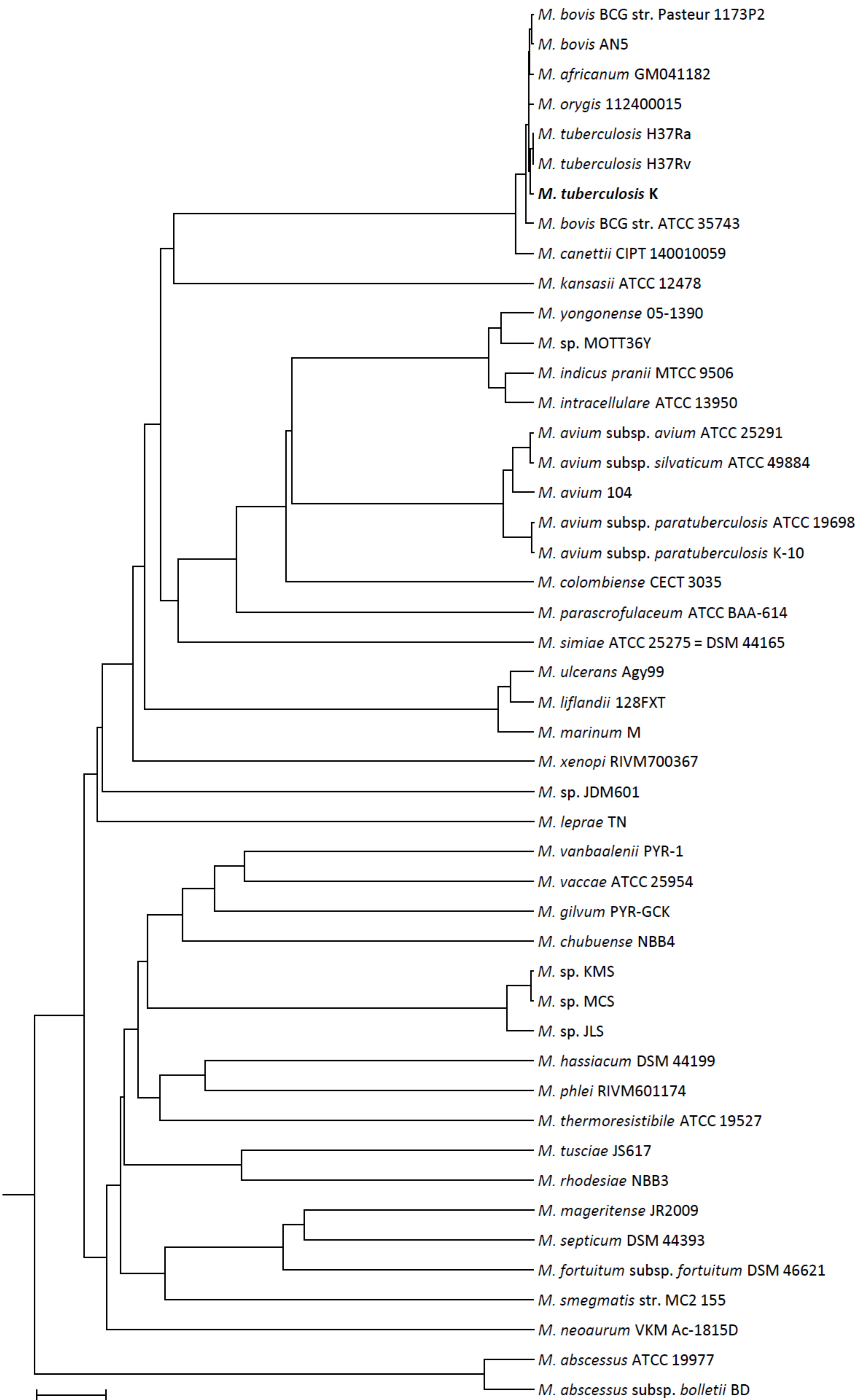

Supplement: Additional file 2: Figure S1. — The genome tree showing the relationships of M. tuberculosis K with other Mycobacterium species based on ANI values. Description of data: To convert the ANI into a distance, its complement to 1 was taken. From this pairwise distance matrix, an ANI tree was constructed using the UPGMA clustering method. (PDF 846 kb) [file 40793_2015_71_MOESM2_ESM.pdf]
